# Alcohol consumption and binge drinking in early pregnancy. A cross-sectional study with data from the Copenhagen Pregnancy Cohort

**DOI:** 10.1186/s12884-015-0757-z

**Published:** 2015-12-08

**Authors:** Mette Langeland Iversen, Nina Olsén Sørensen, Lotte Broberg, Peter Damm, Morten Hedegaard, Ann Tabor, Hanne Kristine Hegaard

**Affiliations:** The Research Unit Women’s and Children’s Health, the Juliane Marie Centre for Women, Children and Reproduction, Copenhagen University Hospital, Rigshospitalet, Copenhagen, Denmark; Clinical Institute of Medicine, Faculty of Health and Medical Sciences, University of Copenhagen, Copenhagen, Denmark; Department of Obstetrics, Rigshospitalet, Copenhagen, Denmark; Center of Fetal Medicine, Department of Obstetrics, Rigshospitalet, Copenhagen, Denmark; Child, Family, and Reproductive Health, Department of Health Science, Faculty of Medicine, Lund University, Lund, Sweden

**Keywords:** Preconception, Pregnancy, Alcohol, Binge drinking, Risk factors, Lifestyle, Reproductive history

## Abstract

**Background:**

Since 2007 the Danish Health and Medicines Authority has advised total alcohol abstinence from the time of trying to conceive and throughout pregnancy. The prevalence of binge drinking among pregnant Danish women has nevertheless been reported to be up to 48 % during early pregnancy. Since the introduction of the recommendation of total abstinence, no studies have examined pre-pregnancy lifestyle and reproductive risk factors associated with this behaviour in a Danish context. The aims of this study were therefore to describe the prevalence of weekly alcohol consumption and binge drinking in early pregnancy among women living in the capital of Denmark. Secondly to identify pre-pregnancy lifestyle and reproductive risk factors associated with binge drinking during early pregnancy.

**Methods:**

Data were collected from September 2012 to August 2013 at the Department of Obstetrics, Rigshospitalet, Copenhagen, Denmark. Self-reported information on each woman’s socio-demographic characteristics, medical history, and lifestyle factors including alcohol habits was obtained from an electronic questionnaire filled out as part of the individual medical record. Descriptive analysis was conducted and multivariate logistic regression analysis was used to assess the potential associated risk factors (adjusted odds ratio (aOR)).

**Results:**

Questionnaires from 3,238 women were included. A majority of 70 %, reported weekly alcohol consumption before pregnancy. The prevalence decreased to 3 % during early pregnancy. The overall proportion of women reporting binge drinking during early pregnancy was 35 % (*n =* 1,134). The following independent risk factors for binge drinking in early pregnancy were identified: lower degree of planned pregnancy, smoking and alcohol habits before pregnancy ((1 unit/weekly aOR 4.48, CI: 3.14 - 6.40), (2–7 units aOR 10.23, CI: 7.44-14.06), (≥8 units aOR 33.18, CI: 19.53-56.36)). Multiparity and the use of assisted reproductive technology were associated with lower odds of binge drinking in early pregnancy.

**Conclusion:**

The prevalence of weekly alcohol consumption decreased considerably during early pregnancy compared with pre-pregnancy levels. Nevertheless one third of the pregnant women engaged in binge drinking. Identification of risk factors for this behaviour renders it possible not only to design prevention strategies, but also to target those most at risk.

**Electronic supplementary material:**

The online version of this article (doi:10.1186/s12884-015-0757-z) contains supplementary material, which is available to authorized users.

## Background

Danish women have one of the highest frequencies of alcohol consumption among the Nordic countries [1]. In recent decades several studies on pregnancy and binge drinking - defined as consumption of five or more alcoholic beverages on the same occasion (one unit is defined as 12 grams of pure alcohol) [2] - have highlighted this area of research. In line with this, previous Danish studies have reported binge drinking prevalences of 24–48 % during early pregnancy [3–5] and this development should be continually followed.

The effect of low-to-moderate levels of alcohol on the fetus is debated, but there is strong evidence to support the association between heavy fetal exposure and adverse events such as preeclampsia, small gestational infant [6] and fetal alcohol spectrum disorder [7, 8]. While these effects are due to continuous exposure, a new emphasis is being put on binge drinking and the risk to the fetus due to peak alcohol exposure. The long-term effect of binge drinking on the neurobehavioral development of the offspring is debated. A cohort study from the Danish National Birth Cohort did not find consistent association between binge drinking and cognitive processes such as planning, organisation, and self-control at 5 years of age [9]. On the other hand, studies have found that binge alcohol exposure in the first weeks of pregnancy may predict difficult temperament and sleeping problems during infancy [10]. Furthermore, an association with learning difficulties and emotional development in childhood has been identified [11–13] as have an increased risk of antisocial personality traits and disorders in adulthood [14]. A recent meta-analysis using data from multiple studies stresses the importance of abstaining from binge drinking during pregnancy based on results showing borderline significant association between binge drinking and impaired child cognition at age 6 months to 14 years [15].

With regard to birth outcomes such as preterm birth [16, 17] no association with binge drinking has been found, while the risk of stillbirth and infant mortality [18, 19] has been documented.

In 2007 the Danish Health and Medicines Authority changed the national guidelines to recommend total abstinence from alcohol for women trying to conceive and during pregnancy [20]. This is in line with many other European countries and based on a precautionary principle. No Danish studies have since then determined the prevalence of alcohol consumption among pregnant women nor identified those at risk of non-compliance. This is of importance because prevalences are not static and will vary over time with changing social norms and official policies.

Risk factors associated with drinking during pregnancy have been identified in international studies and include e.g. higher maternal age [21], smoking [6, 22], unintended pregnancy and binge drinking before conception [22]. To our knowledge however, no studies have investigated a range of reproductive characteristics with the risk of binge drinking including degree of pregnancy planning, time to pregnancy or use of assisted reproductive technology (ART) although women with ART have frequent contact with health care professionals. Additionally a low degree of pregnancy planning negatively influences the lifestyle related to pregnancy, such as lacking intake of folic acid supplement [23]. Knowledge of the pre-pregnancy factors associated with binge drinking could be of importance in the detection of women at risk of binge drinking as well as influence the organization of Danish health care.

The aims of this study were to describe the prevalence of weekly alcohol consumption and binge drinking in early pregnancy among women living in the capital of Denmark, and to identify pre-pregnancy lifestyle and reproductive risk factors associated with binge drinking during early pregnancy.

## Methods

### Study design and population

This study used data from the Copenhagen Pregnancy Cohort, a cohort of pregnant women attending the Department of Obstetrics, Rigshospitalet, University of Copenhagen, Denmark. The hospital serves both as a primary birth facility for Copenhagen city as well as a tertiary referral centre with a total of 6,236 deliveries in 2012 corresponding to approximately 10 % of all births in Denmark. Women with known alcohol use disorder are referred to a special care unit at another hospital in the Capital Region and therefore not included in this cohort.

All pregnant women who scheduled an appointment for their first trimester nuchal translucency scan in the period September 24^th^ 2012 to August 12^th^ 2013 were eligible to be part of this study. A nuchal translucency scan is offered to all pregnant women as part of the national prenatal screening program and in 2012 93 % of all Danish women attended this scan [24].

These women received an email with a link to a clinical questionnaire available in both Danish and English and on average the women responded at 10 gestational weeks (interquartile range 8.7-11.4). Data were collected on socio-demographic characteristics, reproductive and obstetric history, general health status, medication, intake of dietary supplements, pre-pregnancy body mass index (BMI) and lifestyle factors before and during current pregnancy (see Additional file 1). The information was routinely transferred to the pregnant women’s medical records as well as to a research database.

The study was approved by the Danish Data Agency (no. 2007-58-0015). In accordance with Danish legislation, approval from the Ethics Committee was not required. Informed consent was not obtained, as the study is a quality improvement project, which according to the Danish Health Authority recommendations valid at the time of implementation, did not require informed consent.

A total of 4,967 women received a link to the clinical questionnaire. After excluding women who miscarried before returning the questionnaire (*n =* 255), moved outside the municipality (*n =* 57), or had maternity service at another hospital (*n =* 39), 4,616 women remained. Out of these women a total of 4,031 responded to the questionnaire corresponding to a response rate of 87 %. With regard to the specific question on binge drinking 667 did not answer the question, and 126 did not remember (equivalent to 20 %), leaving 3,238 women for our analysis.

### Questionnaire and study variables

The questionnaire has been critically evaluated by health care professionals and thereafter pilot tested on 200 pregnant women before use in our clinical setting. Questions on weekly alcohol consumption were phrased: *How many drinks did you approximately have per week before you became pregnant (one drink is the equivalent of one bottle of beer, one glass of wine, or 4 cl. spirits)?* and *How many drinks do you currently have per week now that you are pregnant?* Women were also asked specifically about binge drinking during pregnancy by: *How many episodes have you had of drinking 5 or more units on a single occasion?* In the questionnaire it was emphasised that this might also include the period before the pregnancy was recognized. The definition of a drink was in accordance with that put forward by the Danish Health and Medicines Authority, i.e. one standard drink is equal to 12 grams of pure alcohol and binge drinking is defined as consumption of five or more alcoholic beverages on the same occasion [2].

Maternal characteristics in the study population were categorized as seen in Table 1. The answers regarding binge drinking were classified yes/no as well as by number of episodes (0, 1, 2 and ≥ 3). Units of weekly alcohol consumption during pregnancy were categorized in three classifications (0, 1 and ≥ 2). The following covariates were also derived from the electronic questionnaire and decided to be included based on a review of the literature before making the analysis: parity, previous miscarriage, time to pregnancy, pregnancy planning, ART, exercise, alcohol before pregnancy and smoking before pregnancy. Parity was categorized as primipara (first pregnancy) or multipara (second or more pregnancy) and previous miscarriage (yes/no). Time to pregnancy was categorised in three intervals in months (0–2, 3–11 and ≥ 12) and pregnancy planning was divided into five response options on a Likert scale [23, 25]: very planned, fairly planned, neither planned or unplanned, fairly unplanned, very unplanned; ART (yes/no), where ART included treatment with hormones, *in vitro* fertilization (IVF), intra-cytoplasmic sperm injection (ICSI), insemination, sperm donation, operation or treatment with frozen eggs. Exercise (yes/no) and smoking (yes/no) represented pre-pregnancy lifestyle factors. Weekly alcohol consumption before pregnancy was categorized in units pr. week (0, 1, 2–7 and ≥ 8) in accordance with the Danish guidelines on low risk alcohol consumption for non-pregnant women, which recommend intake of less than 8 units per week [26].

**Table 1:** Maternal characteristics in the study population, *n* = 3238

Characteristics	Total, *n* (%)
Maternal age (years)	
<24	97 (3)
25-29	774 (24)
30-34	1363 (42)
35-39	789 (24)
≥40	215 (7)
Mean (SD)	32 (5)
*Missing data; 0*	
Parity	
Nulliparous	1960 (61)
Multiparous	1278 (39)
*Missing data. O*	
Danish language skills	
Yes	3068 (96)
No	132 (4)
*Missing data: 38*	
Cohabitation	
Yes	2949 (93)
No	236 (7)
*Missing data; 53*	
Education	
Compulsory school	194 (7)
Skilled	106 (4)
Tertitary	210 (7)
Bachelor or equivalent	897 (31)
Master or equivalent	1451 (51)
*Missing data: 380*	
Occupation	
Working	2066 (71)
Unemployed	166 (6)
Student	464 (16)
Other^a^	206 (7)
*Missing data: 336*	
BMI (kg/m^2^)	
Underweight (<18.5)	154 (5)
Normal (18.5-24.9)	2291 (77)
Overweight (25–29.9)	398 (14)
Obese (>30)	126 (4)
Mean (SD)	23 (4)
*Missing data: 269*	
Chronic illness	
Yes	272 (9)
No	2399 (82)
Other illness^b^	255 (9)
*Missing data: 312*	

^a^ Including stay at home, pensioners, maternity leave

^b ^E.g.: asthma, polycystic ovary syndrome, migraine

The covariates ‘Time to pregnancy’ and ‘Pregnancy planning’ were included as it was hypothesized that the time period elapsing from the earliest attempt to conceive to actual pregnancy, and the degree of planning pregnancy, would influence the women’s drinking patterns. The same argumentation prevailed for inclusion of ART.

### Statistical analysis

We calculated the prevalence of reported binge drinking as well as weekly alcohol consumption before and in early pregnancy. To examine the association between lifestyle factors and reproductive history with binge drinking we performed univariate and multivariate logistic regression analysis. The univariate analysis included all the selected covariates and all were mutually adjusted in the multivariable logistic regression analysis. A priori maternal age, educational level and occupational status were considered potential confounders due to findings from earlier studies [18, 19] and therefore also included in the multivariate logistic analysis. Association was presented as unadjusted odds ratio (OR) and adjusted odds ratio (aOR) with 95 % confidence interval (CI). All statistical analyses were performed in SPSS 20.0 software (IBM).

## Results

### Maternal characteristics in the study population

The women in the Copenhagen Pregnancy Cohort had a mean age of 32 years (SD 5) and 82 % were healthy with no chronic disease, 93 % were living with a partner and 61 % were nulliparous. The majority spoke and wrote Danish (96 %) and nearly half had an academic degree (51 %). The mean pre-pregnancy BMI was 23 kg/m^2^ (SD 4) and 77 % of the women had normal BMI (Table 1).

### Prevalence of weekly alcohol consumption and risk factors for binge drinking

More than 70 % (*n =* 2,265) had weekly alcohol consumption before they became pregnant, with 18 % reporting consumption of 1 unit/week (*n =* 580), 47 % 2–7 units/week (*n =* 1507) while 6 % consumed ≥ 8 units/week (*n =* 178). During early pregnancy the proportion of women with weekly alcohol consumption was around 3 % with 2.6 % consuming one unit weekly (*n =* 86) while less than 1 % consumed two or more units weekly (*n =* 18). The overall proportion of women reporting binge drinking during early pregnancy was 35 % (*n =* 1134). More specifically 20.6 % (*n =* 669) reported one episode, 8.6 % (*n =* 278) reported two episodes and 5.8 % (*n =* 187) reported ≥ 3 episodes at the time of completing the questionnaire.

The results of the univariate and multivariate logistic regression analyses are shown in Table 2.

**Table 2:** Univariate and multivariate associations of reproductive history and lifestyle factors with binge drinking in early pregnancy, *n* = 3,238

Binge drinking	Yes,	No,	crude OR	ajusted OR^a^
	*n* (%)	*n* (%)		95 % Cl
Parity				
Nullipara	820 (42)	1140 (58)	Ref.	Ref.
Multipara	314 (25)	964 (75)	0.45	0.50 (0.39-0.62)
*Missing data: 0*				
Previous miscarriages				
Yes	247 (38)	407 (62)	1.16	0.81 (0.63-1.03)
No	887 (34)	1697 (66)	Ref.	Ref.
*Missing data: 0*				
Time to pregnancy, months				
0-2	676 (40)	1032 (60)	Ref.	Ref.
3-11	338 (36)	597 (64)	0.86	1.00 (0.79-1.26)
≥12	77 (18)	351 (82)	0.34	1.03 (0.68-1.57)
*Missing data: 167*				
Pregnancy planning				
Very planned	362 (25)	1100 (75)	Ref.	Ref.
Fairly planned	386 (44)	499 (56)	2.35	1.78 (1.40-2.25)
Neither planned nor unplanned	214 (44)	272 (56)	2.39	2.74 (2.01-3.73)
Fairly unplanned	62 (53)	54 (47)	3.49	3.02 (1.78-5.10)
Very unplanned *Missing data: 155*	66 (49)	68 (51)	2.95	2.87 (1.70-4.84)
Assisted reproductive technology (ART)				
Yes	32 (8)	370 (92)	0.13	0.12 (0.07-0.21)
No	1051 (39)	1621 (61)	Ref.	Ref.
* Missing data: 164*				
Exercise				
Yes	760 (38)	1269 (62)	1.53	1.12 (0.89-1.43)
No	242 (28)	617 (72)	Ref.	Ref.
* Missing data: 350*				
Alcohol before pregnancy, no of units/week				
0	96 (10)	864 (90)	Ref.	Ref.
1	169 (29)	411 (71)	3.70	4.48 (3.14-6.40)
2-7	727 (48)	780 (52)	8.39	10.23 (7.44-14.06)
≥8	135 (76)	43 (24)	28.26	33.18 (19.53-56.36)
*Missing data: 13*				
Smoking before pregnancy				
No	951 (33)	1943 (67)	Ref.	Ref.
Yes	167 (54)	142 (46)	2.40	2.24 (1.59-3.15)
* Missing data: 230*				

^a^Adjusted for sociodemographic variable: age, education and occupation

After mutual adjustment for the variables in the multivariable logistic regression analysis the groups of women categorizing their pregnancy with a lower degree of planning or who were smokers before pregnancy had statistically significant increased odds of binge drinking. Alcohol consumption before pregnancy also gave statistically significant higher odds for binge drinking as women who reported drinking 1 unit/week had aOR 4.48 (CI: 3.14 - 6.40), women reported drinking 2–7 units/week had aOR 10.23 (CI: 7.44-14.06) and those reported ≥ 8 units/week had aOR 33.18 (CI: 19.53-56.36) compared to women without drinking before pregnancy. Statistically significant lower odds for binge drinking were found for multiparous women with aOR 0.50 (CI: 0.39-0.62) and those pregnant following ART with aOR 0.12 (CI: 0.07-0.21) (Table 2).

When comparing women who did not respond to the question on binge drinking to those who responded we found that non-responders differed by having weekly alcohol consumption prior to pregnancy (*p <* 0.001), a lower degree of pregnancy planning (*p <* 0.001), a lower proportion of women receiving ART (*p =* 0.039) and shorter time to pregnancy (*p =* 0.031). Non-responders were comparable on all other counts (Table 3).

**Table 3:** Responders and non-responders^a^ to the question about binge drinking

*Characteristics*	*Responding, n (%)*	*Missing, n (%)*	*P-value*
*Maternal age (years)*			
*<24*	*97 (3)*	*37 (5)*	
*25-29*	*774 (24)*	*208 (26)*	
*30-34*	*1363 (42)*	*311 (39)*	
*35-39*	*789 (24)*	*187 (24)*	
*≥40*	*215 (7)*	*50 (6)*	*0.081*
*Parity*			
*Nulliparous*	*1960 (61)*	*499 (63)*	
*Multiparous*	*1278 (39)*	*294 (37)*	*0.215*
*Danish language skills*			
*Yes*	*3068 (96)*	*755 (96)*	
*No*	*132 (4)*	*28 (4)*	*0.483*
*Cohabitation*			
*Yes*	*2949 (93)*	*716 (92)*	
*No*	*236 (7)*	*63 (8)*	*0.521*
*Education*			
*Compulsory school*	*194 (7)*	*56 (8)*	
*Skilled*	*106 (4)*	*23 (3)*	
*Tertitary*	*210 (7)*	*48 (7)*	
*Bachelor or equivalent*	*897 (31)*	*220 (34)*	
*Master or equivalent*	*1451 (51)*	*308 (47)*	*0.321*
*Occupation*			
*Working*	*2066 (71)*	*454 (68)*	
*Unemployed*	*166 (6)*	*41 (6)*	
*Student*	*464 (16)*	*129 (19)*	
*Other* ^*b*^	*206 (7)*	*46 (7)*	*0.204*
*BMI (kg/m* ^*2*^ *)*			
*Underweight (<18.5)*	*154 (5)*	*35 (5)*	
* Normal (18.5-24.9)*	*2291 (77)*	*518 (76)*	
* Overweight (25–29.9)*	*398 (14)*	*93 (14)*	
* Obese (>30)*	*126 (4)*	*36 (5)*	*0.689*
* Mean (SD)*	*23 (4)*		
*Chronic illness*			
* Yes*	*272 (9)*	*64 (9)*	
*No*	*2399 (82)*	*594 (84)*	
*Other illness* ^*c*^	*255 (9)*	*51 (7)*	*0.399*
*Miscarriage*			
*Yes*	*654 (20)*	*150 (19)*	
*No*	*2584 (80)*	*643 (81)*	*0.418*
*Time to pregnancy, months*			
*0-2*	*1708 (56)*	*434 (61)*	
*3-11*	*935 (30)*	*195 (27)*	
*≥12*	*428 (14)*	*83 (12)*	*0.031*
*Pregnancy planning*			
*Very planned*	*1462 (47)*	*288 (40)*	
*Fairly planned*	*885 (29)*	*202 (28)*	
*Neither planned nor unplanned*	*486 (16)*	*160 (22)*	
*Fairly unplanned*	*116 (4)*	*38 (5)*	
*Very unplanned*	*134 (4)*	*34 (5)*	*0.000*
*Assisted reproductive technology (ART)*			
*Yes*	*402 (13)*	*74 (10)*	
*No*	*2672 (87)*	*648 (90)*	*0.039*
*Exercice*			
*Yes*	*2019 (70)*	*475 (71)*	
*No*	*859 (30)*	*189 (28)*	*0.514*
*Alcohol before pregnancy, no of units/week*			
*0*	*960 (30)*	*17 (13)*	
*1*	*580 (18)*	*23 (18)*	
*2-7*	*1507 (47)*	*74 (58)*	
*≥8*	*178 (5)*	*14 (11)*	*0.000*
*Smoking before pregnancy*			
*Yes*	*309 (10)*	*87 (11)*	
*No*	*2894 (90)*	*692 (89)*	*0.203*

^a ^Including those who answered ‘don’t know’

^b^ Including stay at home, pensioners, maternity leave

^c^ E.g: asthma, polycystic ovary syndrome, migraine

## Discussion

In this Danish study nearly three quarters of the women reported weekly alcohol consumption before pregnancy, with a major decline to around 3 % during early pregnancy. Around one third of the women reported binge drinking during early pregnancy. Risk factors for binge drinking were: lower degree of pregnancy planning, smoking as well as weekly alcohol consumption before pregnancy. Heavy weekly alcohol consumption before pregnancy (≥8 units/week) was the variable with the highest odds estimate for binge drinking (aOR 32.63, CI: 19.23-55.36). Multiparity or pregnant following ART were associated with lower odds of binge drinking in early pregnancy.

### Comparison with existing literature

The fact that more than 70 % reported weekly alcohol consumption before pregnancy clearly illustrates that women generally do not comply with national guidelines. This percentage is actually lower than newly reported levels of weekly alcohol consumption among Danish women of fertile age [5], this in spite of the fact that since 2007 these guidelines have clearly recommended total alcohol abstinence while trying to conceive and during pregnancy. However, it is slightly lower than reported levels of 80 to 89 % found in neighboring Sweden as well as in Australia and New Zealand [27–30]. The decline in weekly alcohol consumption during early pregnancy to 3 % in the Copenhagen Pregnancy Cohort is in accordance with contemporary literature, which also reports decreasing intake after confirmed pregnancy with a prevalence of approximately 5 % [23, 31].

While the proportion of women in the Copenhagen Pregnancy Cohort who engaged in one or more binge drinking episodes in 2012–13 was 35 %, another Danish study reported a prevalence of 22 % in early pregnancy with data from 2011 [23]. Data from a large regional cohort collected in the second largest city of Denmark (*n =* 56.545) in 2013 revealed that 40 % of the women included around gestational week 12 reported one or more episodes of binge drinking [5]. These different prevalences may be due to a diverse age distribution and socio-economic setting in the populations studied. Interestingly, the prevalence of binge drinking in early pregnancy presented in international literature ranges from 3 % to 55 % [22, 30–32] but is for the most part lower than the Danish figures. These differing binge drinking levels may indicate that it is difficult to generalize between populations, and prevalence should be understood in the regional context of the respective study.

The general Danish population has one of the highest levels of alcohol consumption in Scandinavia [33] and alcohol is widely accepted and normalized in Denmark [34]. It is likely that lifestyle prior to conception is carried into pregnancy. Perhaps the relatively high Danish level of weekly alcohol consumption before pregnancy and binge drinking during early pregnancy reflect an influence of the national alcohol culture on pregnant women as well as on those contemplating pregnancy.

An Australian qualitative study supports this notion. In semi-structured interviews 12 women expressed the dilemma of not wanting to reveal an early pregnancy and at the same time engaging in social events that included alcohol consumption. Furthermore the study found that women lacked knowledge about the potential harmful effects of alcohol on the fetus [35]. Similarly, the social norm as well as a lack of knowledge may partly explain why women in the Copenhagen Pregnancy Cohort engage in alcohol consumption.

We have, along with previous research, investigated risk factors for binge drinking and non-compliance with general recommendations. Strandberg-Larsen et al. examined binge drinking in the early and unrecognized part of pregnancy and found this was more common among nulliparous women, age group 25–29 years and in those with higher degrees of education [4]. Our findings confirm these results with regards to parity, but not age and education. Furthermore our study along with others has established that smoking [31] and weekly alcohol consumption before pregnancy are strongly associated with continued drinking in early pregnancy [4, 22, 27, 36].

Another finding is that a lower degree of pregnancy planning is associated with binge drinking. This is in accordance with prior Danish results indicating that women with unintended pregnancies more often engage in binge drinking during early pregnancy than women with a very planned pregnancy [4, 23]. The association between a higher degree of pregnancy planning and lower rates of binge drinking is not unexpected given that compliance with overall guidelines before and during pregnancy is positively influenced by higher degree of pregnancy planning [23].

We also found that being pregnant after ART reduced the odds of binge drinking. Perhaps this is due to a higher level of knowledge about the effects of alcohol because of information given at the fertility clinic or because these pregnancies are very well planned and this group is highly motivated to change lifestyle in order to achieve pregnancy. To our knowledge no other studies have examined the association between pregnancy after ART and binge drinking.

### Strengths and limitations

Strengths of this study are the large sample size compared to previous studies [3, 29] and the fact that the hospital serves as a primary birth facility and is therefore representative for women in Denmark’s capital Copenhagen. This is largely due to the recruitment strategy of inviting women to participate when booking their first trimester nuchal translucency scan – a part of the routine Danish antenatal screening program. A further strength is the recentness of the data (2012–2013), which must be interpreted to reflect the current pattern of alcohol consumption. In addition our questionnaire was translated into English and as a result we included women not understanding Danish, which was 4 % in our study population. In earlier Danish studies [4, 37] good Danish speaking skills have often been inclusion criteria, which may have led to an underrepresentation of non-Danish speaking women. Although we made an effort to minimize recall bias by obtaining information on alcohol consumption early in pregnancy [38], we acknowledge that the risk of reporting bias is a potential limitation. It is plausible that pregnant women underreport alcohol habits [29, 38] and this may also be the case in our study. The missing data on binge drinking (*n =* 793) may be an indication that these questions are of a sensitive nature and subject to social-desirability bias [35, 39]. With regard to the key question on binge drinking, non-responders were characterized by having weekly alcohol consumption before pregnancy (*p <* 0.001) and a lower degree of planned pregnancy (*p <* 0.001) compared to those who answered the question. Given that weekly alcohol consumption and a lower degree of pregnancy planning were found to be associated with binge drinking this might have led to an underestimation of the prevalence of binge drinking. Furthermore, we are aware of the risk of underestimation of adjusted odds ratios for the associations between weekly alcohol consumption and pregnancy planning with binge drinking. It is difficult, however, to assess whether odds ratios for all other covariates could possibly be affected and whether this is leading to underestimation, overestimation or unchanging estimates.

In our study around 20 % did not respond the question about binge drinking. When the outcome is missing, there are generally no benefits in imputing the outcome, unless there is access to an auxiliary variable that is highly correlated with the outcome but not included in the multivariable model [40]. As we have included all important predictors in the multivariable model, we have no such extra variables that can be used in an imputation model. Actually, using imputed values of the dependent variable does not provide additional information but instead introduces additional error due to simulation error.

Though our questionnaire has been pilot-tested it can be discussed whether the lack of psychometric validation for the variable “degree of pregnancy planning” is a limitation in our study. Methods for measuring pregnancy planning have varied widely between studies and each has different merits and shortcomings [41]. Our finding that pregnancy planning is associated with binge drinking is in accordance with studies both by Backhausen et al. [23] and Tyden et al. [25]. Backhausen utilized the multi-itemed London Measure of Unplanned Pregnancies that has undergone validation [42].

The way of phrasing the questions should also be taken into consideration when describing drinking patterns of pregnant women. Furthermore, some studies have calculated separate rates for binge drinking before and after recognized pregnancy respectively [4, 22, 27]; we did not. Women in the Copenhagen Pregnancy Cohort were encouraged to recall the time before they knew of their pregnancy, but were not specifically asked to distinguish between the period before and after, respectively, of knowledge of their pregnancy. If women did indeed engage in binge episodes because they were unaware of their pregnancy as suggested in previous Danish studies [4, 5], this may be a potential limitation in our study. Such a distinction may be advantageous in future studies.

While there is no gold standard for collection of data on alcohol consumption, questionnaires and interviews have been found to provide comparable data [43]. A self-administered questionnaire, which was our choice, is acknowledged to be a good method for obtaining information on lifestyles related to less socially acceptable behaviors such as drinking and smoking [43].

### Implications

The findings from our study may indicate a need for enhanced preconception care because of the marked differences between Danish national recommendations on alcohol abstinence in relation to conception and during early pregnancy and actual habits among women. The results emphasize that information should target not only pregnant women but also those intending pregnancy. This may have a positive effect on alcohol consumption in the unrecognized and early part of pregnancy. Furthermore, our study identified certain characteristics in pregnant women who engage in binge drinking and with this knowledge it is possible not only to design prevention strategies but also to target those women most at risk. Future clinical intervention studies are needed to examine the effect of such targeted prevention strategies in the preconception period on the rates of binge drinking.

## Conclusion

More than seventy percent of women in the Copenhagen Pregnancy Cohort reported weekly alcohol consumption in the period before pregnancy. Although most of these women had changed behavior at the time of answering the questionnaire, this study also finds that more than one third of the pregnant women report binge drinking in early pregnancy. While this may be because the pregnancy is not yet recognized, it remains a matter of particular concern and contrary to national recommendations on alcohol. This study found the factors associated with binge drinking to be: low degree of pregnancy planning, smoking, and alcohol habits before pregnancy. The study therefore emphasizes a need for information and enhanced preconception care to address alcohol habits in early pregnancy.

## Additional file

Additional file 1:
**Questionnaire**. (PDF 823 kb)

## Abbreviations

aOR: adjusted odds ratio
ART: assisted reproductive technology
BMI: body mass index
CI: confidence interval
ICSI: intra-cytoplasmic sperm injection
IVF: *in vitro* fertilization
OR: odds ratio
SD: standard deviation

## References

[1] Mäkelä P, Fonager K, Hibell B, Nordlund S, Sabroe S, Simpura J (2001). Episodic heavy drinking in four Nordic countries: a comparative survey. Addiction.

[2] Sundhedsstyrelsen (2013). Danskernes sundhed. Den nationale sundhedsprofil.

[3] Kesmodel U (2001). Binge drinking in pregnancy--frequency and methodology. Am J Epidemiol.

[4] Strandberg-Larsen K, Rod Nielsen N, Nybo Andersen A-M, Olsen J, Grønbaek M (2008). Characteristics of women who binge drink before and after they become aware of their pregnancy. Eur J Epidemiol.

[5] Petersen G, Kesmodel U, Strandberg-Larsen K on behalf of Sundhedsstyrelsen (2015). Alcohol consumption among pregnant women and women of childbearing age in Denmark.

[6] Salihu HM, Kornosky JL, Lynch O, Alio AP, August EM, Marty PJ (2011). Impact of prenatal alcohol consumption on placenta-associated syndromes. Alcohol.

[7] Jones KL, Smith DW, Ulleland CN, Streissguth P (1973). Pattern of malformation in offspring of chronic alcoholic mothers. Lancet.

[8] Mattson SN, Schoenfeld AM, Riley EP (2001). Teratogenic effects of alcohol on brain and behavior. Alcohol Res Health.

[9] Skogerbø A, Kesmodel U, Wimberly T, Støvring H, Bertrand J, Landrø N (2012). The effects of low to moderate alcohol consumption and binge drinking in early pregnancy on executive function in 5-year-old children. BJOG.

[10] Alvik A, Torgersen AM, Aalen OO, Lindemann R (2011). Binge alcohol exposure once a week in early pregnancy predicts temperament and sleeping problems in the infant. Early Hum Dev.

[11] Olson HC, Streissguth AP, Sampson PD, Barr HM, Bookstein FL, Thiede K (1997). Association of prenatal alcohol exposure with behavioral and learning problems in early adolescence. J Am Acad Child Adolesc Psychiatry.

[12] Niclasen J, Andersen A-MN, Strandberg-Larsen K, Teasdale TW (2014). Is alcohol binge drinking in early and late pregnancy associated with behavioural and emotional development at age 7 years?. Eur Child Adolesc Psychiatry.

[13] Streissguth AP, Barr HM, Sampson PD (1990). Moderate prenatal alcohol exposure: effects on child IQ and learning problems at age 7 1/2 years. Alcohol Clin Exp Res.

[14] Barr HM, Bookstein FL, O’Malley KD, Connor PD, Huggins JE, Streissguth AP (2006). Binge drinking during pregnancy as a predictor of psychiatric disorders on the Structured Clinical Interview for DSM-IV in young adult offspring. Am J Psychiatry.

[15] Flak AL, Su S, Bertrand J, Denny CH, Kesmodel US, Cogswell ME (2014). The association of mild, moderate, and binge prenatal alcohol exposure and child neuropsychological outcomes: a meta-analysis. Alcohol Clin Exp Res.

[16] Meyer-Leu Y, Lemola S, Daeppen J-B, Deriaz O, Gerber S (2011). Association of moderate alcohol use and binge drinking during pregnancy with neonatal health. Alcohol Clin Exp Res.

[17] Cooper DL, Petherick ES, Wright J (2013). The association between binge drinking and birth outcomes: results from the Born in Bradford cohort study. J Epidemiol Community Health.

[18] Strandberg-Larsen K, Nielsen NR, Grønbaek M, Andersen PK, Olsen J, Andersen A-MN (2008). Binge drinking in pregnancy and risk of fetal death. Obstet Gynecol.

[19] Strandberg-Larsen K, Grønboek M, Andersen A-MN, Andersen PK, Olsen J (2009). Alcohol drinking pattern during pregnancy and risk of infant mortality. Epidemiology.

[20] Brot C, Poulsen, Annette, Danmark, Sundhedsstyrelsen (2009). Anbefalinger for svangreomsorgen. Kbh.: Sundhedsstyrelsen : [eksp.] Komiteen for Sundhedsoplysning.

[21] Murphy DJ, Mullally A, Cleary BJ, Fahey T, Barry J (2013). Behavioural change in relation to alcohol exposure in early pregnancy and impact on perinatal outcomes--a prospective cohort study. BMC Pregnancy Childbirth.

[22] Ethen MK, Ramadhani TA, Scheuerle AE, Canfield MA, Wyszynski DF, Druschel CM (2009). Alcohol consumption by women before and during pregnancy. Matern Child Health J.

[23] Backhausen MG, Ekstrand M, Tydén T, Magnussen BK, Shawe J, Stern J (2014). Pregnancy planning and lifestyle prior to conception and during early pregnancy among Danish women. Eur J Contracept Reprod Health Care.

[24] Frøslev PA, Carlsen K. National database for føtalmedicin-FØTO Databasen. National annual report. 2012;2012.

[25] Tydén T, Stern J, Nydahl M, Berglund A, Larsson M, Rosenblad A (2011). Pregnancy planning in Sweden--a pilot study among 270 women attending antenatal clinics. Acta Obstet Gynecol Scand.

[26] Sundhedsstyrelsen. Sundhedsstyrelsens nye udmelding vedrørende alkohol. Homepage: http://sundhedsstyrelsen.dk/~/media/58BECF8BA35441FDA84FE76986325728.ashx

[27] Skagerström J, Alehagen S, Häggström-Nordin E, Årestedt K, Nilsen P (2013). Prevalence of alcohol use before and during pregnancy and predictors of drinking during pregnancy: a cross sectional study in Sweden. BMC Public Health.

[28] Peadon E, Payne J, Henley N, D’Antoine H, Bartu A, O’Leary C (2011). Attitudes and behaviour predict women’s intention to drink alcohol during pregnancy: the challenge for health professionals. BMC Public Health.

[29] Alvik A, Haldorsen T, Groholt B, Lindemann R (2006). Alcohol consumption before and during pregnancy comparing concurrent and retrospective reports. Alcohol Clin Exp Res.

[30] Ho R, Jacquemard R (2009). Maternal alcohol use before and during pregnancy among women in Taranaki, New Zealand. N Z Med J.

[31] Gladstone J, Levy M, Nulman I, Koren G (1997). Characteristics of pregnant women who engage in binge alcohol consumption. CMAJ.

[32] Anderson AE, Hure AJ, Forder PM, Powers J, Kay-Lambkin FJ, Loxton DJ (2014). Risky drinking patterns are being continued into pregnancy: a prospective cohort study. PLoS One.

[33] Hibell B, Centralförbundet för alkohol-och narkotikaupplysning, European School Survey Project on Alcohol and Other Drugs (2004). The ESPAD report 2003: alcohol and other drug use among students in 35 European countries.

[34] Grønkjær M, Curtis T, Crespigny CD, Delmar C (2011). Acceptance and expectance: Cultural norms for alcohol use in Denmark. Int J Qual Stud Health Well-being.

[35] Jones SC, Telenta J (2012). What influences Australian women to not drink alcohol during pregnancy?. Aust J Prim Health.

[36] Mallard SR, Connor JL, Houghton LA (2013). Maternal factors associated with heavy periconceptional alcohol intake and drinking following pregnancy recognition: a post-partum survey of New Zealand women. Drug Alcohol Rev.

[37] Kesmodel U, Kesmodel P, Larsen A, Secher N (2003). Use of alcohol and illicit drugs among pregnant Danish women, 1998. Scand J Public Health.

[38] Feldman Y, Koren G, Mattice K, Shear H, Pellegrini E, MacLeod SM (1989). Determinants of recall and recall bias in studying drug and chemical exposure in pregnancy. Teratology.

[39] Welte JW, Russell M (1993). Influence of socially desirable responding in a study of stress and substance abuse. Alcohol Clin Exp Res.

[40] Roderick JA (1992). Little Regression with missing X’s: A review. J Am Stat Assoc.

[41] Petersen R, Moos MK (1997). Defining and measuring unintended pregnancy: issues and concerns. Womens Health Issues.

[42] Barrett G, Smith SC, Wellings K (2004). Conceptualisation, development, and evaluation of a measure of unplanned pregnancy. J Epidemiol Community Health.

[43] Kesmodel U, Olsen SF (2001). Self reported alcohol intake in pregnancy: comparison between four methods. J Epidemiol Community Health.

