# Supplementary material for: Alcohol consumption and binge drinking in early pregnancy. A cross-sectional study with data from the Copenhagen Pregnancy Cohort
Source: BMC Pregnancy Childbirth. 2015 Dec 8;15:327. doi: 10.1186/s12884-015-0757-z (PMC4673744; doi:10.1186/s12884-015-0757-z)
Supplement: Additional file 1: — Questionnaire. (PDF 823 kb) [file 12884_2015_757_MOESM1_ESM.pdf]

Information sheet 1

This schedule was completed on date: ______/______-_________

Personal information

First name(s): ______________________________ Surname:__________________________

Civil registration number (cpr):___________________________

Provide 1 or 2 contact numbers:

Telephone number 1:_________________________ Telephone number 2:__________________

E-mail: ______________________________@________________________

Are you living with a parent/spouse? No ☐ Yes ☐

What is your current civil status? (*Only tick off once*)

Married ☐ Single ☐ Divorced ☐ Widowed ☐ Separated ☐ Civil Partnership ☐

Information

4.1. Can you read and understand Danish? Yes ☐ No ☐

4.2. Do you need an interpreter? Yes ☐ No ☐

4.3. What is your nationality?:____________________________

4.4. What is your native language? (indicate several if necessary):________________________________

4.5. Do you have special needs/concerns, i.e. problems with walking, with your vision or hearing, emotional or other issues Yes ☐ No ☐

4.6. If yes, please indicate:____

____

4.7. Are you in contact with your municipal case officer? Yes ☐ No ☐

4.8. If yes, why?____

This pregnancy

5.1. Has your delivery date been determined through scanning? Yes ☐ No ☐

5.2. If yes, what is the due date from ultrasound scanning: _______/_______-__________

5.3. How long did it take you to become pregnant this time?

Years:_________ Months:_________

5.4. I did not intentionally become pregnant ☐

5.5. In connection with this pregnancy, did you receive infertility treatment e.g. hormones, IVF, ICSI, insemination or operation? Yes ☐ No ☐

5.6. If yes, which treatment?

In vitro-fertilisation/IVF ☐
ICSI ☐
Insemination ☐
Hormone treatment/stimulation ☐
Sperm donation ☐
Operation ☐
Treatment with frozen eggs ☐

5.13. How planned is your current pregnancy? (*Only tick off once*)

Very well planned ☐
Fairly well planned ☐
Neither planned nor planned ☐
Fairly unplanned ☐
Totally unplanned ☐

Previous pregnancies

6.1. Have you been pregnant before (including miscarriages)? Yes ☐ No ☐

If no, go to question 9.1.

If you have been pregnant before, when and how did your pregnancy(cies) end? (*Please only tick off once in every line*)

	**Year**	**Birth**			**Miscarriage**		**Ectopic pregnancy**
		Delivery 1 child	Delivery twins	Delivery triplets	0-3rd months	4-6th months	
1.							
2.							
3.							
4.							
5.							
6.							

Previous births

7.1. Previous deliveries (If you gave birth to twins or triplets please fill out a line for each child)

	Year + Place of birth	Live birth	Sex	Birth weight in grams	More than 3 weeks premature	Caesarean section	Healthy/ normal development
1.		Yes ☐ No☐	Boy ☐ Girl ☐		Yes ☐ No☐	Yes ☐ No☐	Yes ☐ No☐
2.		Yes ☐ No ☐	Boy ☐ Girl ☐		Yes ☐ No☐	Yes ☐ No☐	Yes ☐ No☐
3.		Yes ☐ No ☐	Boy ☐ Girl ☐		Yes ☐ No☐	Yes ☐ No☐	Yes ☐ No☐
4.		Yes ☐ No ☐	Boy ☐ Girl ☐		Yes ☐ No☐	Yes ☐ No☐	Yes ☐ No☐
5.		Yes ☐ No ☐	Boy ☐ Girl ☐		Yes ☐ No☐	Yes ☐ No☐	Yes ☐ No☐
6.		Yes ☐ No☐	Boy ☐ Girl ☐		Yes ☐ No☐	Yes ☐ No☐	Yes ☐ No☐

Previous pregnancies and births – complications or illness

Did you ever rupture your sphincter anus in connection with giving birth?

Yes ☐ No ☐

Have you been diagnosed with pelvic girdle syndrome by a doctor or physiotherapist in a previous pregnancy?

Yes ☐ No ☐

Please indicate whether you have had problems or diseases in a previous pregnancy, birth or postpartum period:

____

____

____

Your health

How would you assess your general state of health before your pregnancy? (*Only tick off once*)

☐ Really good ☐ Good ☐ Alright ☐ Poor ☐ Very poor

Did you have any of the following conditions/diseases when you became pregnant? (*Tick as many as required*)

- ☐ Yes, high blood pressure
- ☐ Yes, lung disease Which is:: ______
- ☐ Yes, diabetes type 1
- ☐ Yes, diabetes type 2
- ☐ Yes, metabolic disorder Which is: ______
- ☐ Yes, kidney disease Which is: ______
- ☐ Yes, epilepsy
- ☐ Yes, arthritis Which is: ______
- ☐ Yes, mental illness Which is: ______
- ☐ Yes, heart disease Which is: ______
- ☐ Other disease Which is:______
- ☐ No, I did not suffer from any diseases

Have you ever consulted a psychologist? Yes ☐ No ☐

Have you ever consulted a psychiatrist? Yes ☐ No ☐

Have you had an operation? No ☐ Yes ☐

If yes, when (year): ______

What was the operation for: ______

______

Does anyone in your family have diabetes (diabetes type 1)? No ☐ Yes ☐

If yes, please specify who: (*Tick as many as required*)

a. ☐ Yes, parents

b. ☐ Yes, grand parents

c. ☐ Yes, siblings

d. ☐ Yes, children

Does anyone in your family have diabetes (diabetes type 2)? No ☐ Yes ☐

If yes, please specify who: (*Tick as many as required*)

a. ☐ Yes, parents

b. ☐ Yes, grandparents

c. ☐ Yes, siblings

d. ☐ Yes, children

Did you take any medicine (including pain relieving medicine) within the last three months before pregnancy?

No ☐ Yes ☐

If yes, please specify which medicine:

	Type of medicine	Frequency		
a.		☐ Daily	☐ 1-2 times a week	☐ Rarely
b.		☐ Daily	☐ 1-2 times a week	☐ Rarely
c.		☐ Daily	☐ 1-2 times a week	☐ Rarely

Do you take any medicine (including pain relieving medicine) now, during your pregnancy?

No ☐ Yes ☐

If yes: please specify which medicine:

	Type of medicine	Frequency		
a.		☐ Daily	☐ 1-2 times a week	☐ Rarely
b.		☐ Daily	☐ 1-2 times a week	☐ Rarely
c.		☐ Daily	☐ 1-2 times a week	☐ Rarely

Well-being

The following questions concern your well-being during the last two weeks

For each of the five statements below, please tick the number which is closest to how you have been feeling during the last two weeks. Note that higher numbers mean better well-being.

Example: If you have felt happy and in good spirits **more than half of the time** during the last two weeks, then tick the box with the number 3.

**"Over the last two weeks..."**	All the time	Most of the time	More than half the time	Less than half the time	Some of the time	At no time
I have felt cheerful and in good spirits	5	4	3	2	1	0
I have felt calm and relaxed	5	4	3	2	1	0
I have felt active and vigorous	5	4	3	2	1	0
I woke up feeling fresh and rested	5	4	3	2	1	0
My daily life has been filled with things that interest me	5	4	3	2	1	0

Weight and height

What did you weigh before you became pregnant?: ______kilos

What is your current weight?: ________kilos

What is your height?: _______cm

Smoking

Did you smoke before you became pregnant? Yes ☐ go to 12.2 No ☐ go to 12.3

12.2. If yes, how much did you smoke on a daily basis? ________ (cigarettes)

12.3. Did you smoke prior to your pregnancy? (*Only tick off once*)

I have never smoked ☐

I stopped smoking because I was planning this pregnancy ☐

I stopped smoking long before I planned this pregnancy ☐

Are you a smoker no? Yes ☐ No ☐

If yes, how much do you smoke on a daily basis? ________(cigarettes)

Did your partner or spouse smoke when you became pregnant? Yes ☐ No ☐

Does your partner or spouse smoke now? Yes ☐ No ☐

Alcohol comsumption

How many units of alcohol (one unit corresponds to 1 beer, 1 glass of wine or 4 cl. of spirits) did you consume per week before you became pregnant? _________ (Number of units)

Approximately how many units of alcohol (one unit corresponds to 1 beer, 1 glass of wine or 4 cl. of spirits) do you drink per week during your pregnancies? _________ (Number of units)

The following question concerns your entire pregnancy, including the first weeks when you were unaware that you were pregnant.

How many times did you drink 5 or more units of alcohol on a single occasion?

Number of times:___________________________

Do not remember/do not know ☐

Other intoxicants

(Tick one box for each question)

14.1. Did you smoke hash during the year prior to your pregnancy? Yes ☐ No ☐

14.2. Do you smoke hash now? Yes ☐ No ☐

14.3. Did you take sedatives during the year prior to your pregnancy? Yes ☐ No ☐

14.4. Do you take sedatives now? Yes ☐ No ☐

14.5. Did you use narcotics (heroine, morphine, ketogan) during the year prior to your pregnancy Yes ☐ No ☐

14.6. Do you use narcotics (heroine, morphine, ketogan) now? Yes ☐ No ☐

14.7. Did you use amphetamine, speed, ecstasy or other drugs during the year prior to your pregnancy? Yes ☐ No ☐

14.8. Do you use amphetamine, speed ecstasy or other drugs now? Yes ☐ No ☐

14.9. Did you use cocaine during the year prior to your pregnancy? Yes ☐ No ☐

14.10. Do you use cocaine now? Yes ☐ No ☐

Food supplements

Did you take a daily vitamin D tablet at the time you became pregnant? Yes ☐ No ☐

If yes, which dosis per day__________

Do you take a daily vitamin D tablet now? Yes ☐ No ☐

If yes, which dosis per day__________

Did you take folic acid daily at the time you became pregnant? Yes ☐ No ☐

Do you take folic acid daily now? Yes ☐ No ☐

Did you take daily multivitamins for pregnant women at the time you became pregnant? Yes ☐ No ☐

Do you take a daily vitamin D tablet now? Yes ☐ No ☐

Do you currently take a daily iron supplement of at least 40-50 mg? Yes ☐ No ☐

Do you drink milk/consume milk products every day? Yes ☐ No ☐

Exercise habits

Did you exercise before you became pregnant? Yes ☐ No ☐

If yes, please indicate type of exercise and hours per week:

20.2. ☐ Running: ☐ (hours/week)
20.3. ☐ Strength training: ☐ (hours/week)
20.4. ☐ Yoga: ☐ (hours/week)
20.5. ☐ Cycling, including cycling to work: ☐ (hours/week)
20.6. ☐ Brisk walking: ☐ (hours/week)
20.7. ☐ Spinning: ☐ (hours/week)
20.8. ☐ Fitness: ☐ (hours/week)
20.9. ☐ Swimming: ☐ (hours/week)
20.10. ☐ Aquatic exercise ☐ (hours/week)
20.11. ☐ Horseback riding ☐ (hours/week)
20.12. ☐ Other type of exercise ☐ (hours/week)

If other, please indicate:______________________

Are you currently exercising? Yes ☐ No ☐

If yes, please indicate type of exercise and hours per week:

20.15. ☐ Running: ☐ (hours/week)
20.16. ☐ Strength training: ☐ (hours/week)
20.17. ☐ Yoga: ☐ (hours/week)
20.18. ☐ Cycling, including cycling to work: ☐ (hours/week)
20.19. ☐ Brisk walking: ☐ (hours/week)
20.20. ☐ Spinning: ☐ (hours/week)
20.21. ☐ Fitness: ☐ (hours/week)
20.22. ☐ Swimming: ☐ (hours/week)
20.23. ☐ Aquatic exercise ☐ (hours/week)
20.24. ☐ Horseback riding ☐ (hours/week)
20.25. ☐ Other type of exercise ☐ (hours/week)

If other, please indicate:______________________

Worry during pregnancy

The following are issues that may cause worry during pregnancy. Many pregnant women experience worry. We wish to know if you are worried about any of the following issues. For each of the them, please tick the number that corresponds to the current level of your worry (one tick per line).

	Not a worry<br>0	1	2	3	4	Major worry<br>5
21.1. Your housing						
21.2. Money problems						
21.3. Your relationship with your husband/partner						
21.4. Your relationship with your family and friends						
21.5. Your own health						
21.6. The health of someone close to						

you						
21.7. Employment problems						
21.8. The possibility of something being wrong with the baby						
21.9. Going to hospital						
21.10. Internal examinations						
21.11. Giving birth						
21.12. Coping with the new baby						
21.13. Giving up work						
21.14. Whether your partner will be there for you for the birth						
21.15. The possibility that the birth starts too early						
21.16. The possibility of miscarriage						

21.17. Do you have other worries? No ☐ Yes ☐

21.18 If yes, please describe which:______________________________

____

____

Education and employment status

At what level did you complete your education? (*Only tick off once*)

7th-9th grade.. ☐

10th-11th grade…………………………………………… ☐

Baccalaureate (including HF, HTX, HHX)...................... ☐

What educational training did you complete?

None... ☐

Technical degree……………………………………… ☐

Short degree (1-2 years)………………………………. ☐
Intermediate degree (3-4 years)……………………… ☐
Higher academic degree... ☐

What is your current employment status?

Employed.. ☐
Unemployed…...............…………………………… ☐
Housewife……………………………………........ ☐
On pension…………………………………………… ☐
On welfare………………………………………........ ☐
Student…………………………………..................... ☐
Maternity leave……………………………................. ☐
Other………………….. ……………………………… ☐
If other, please indicate:________________________________

What is/was your job? (Give a precise indication, e.g. ‘hospital laboratory technician’ not just ‘technician’; ‘primary school teacher’ not just ‘teacher’ etc.): ___________________________________

How long is your working week?_____________________ (hours)

What hours do you work?

Between 7 a.m. and 5 p.m. .... ☐
Early mornings……………………………………....... ☐
Evenings……………………………………………......... ☐
Nights…………………………………………….......... ☐
Shift work……...................................………………..... ☐

Work absence / Working conditions

Have you been absent from work due to illness or for other reasons during this pregnancy?

No ☐ Yes ☐

If yes, please indicate the reason (*tick several as required*):

Because of pregnancy-related discomfort/complications ☐

If yes, which?:_______________________________________

Number of days absent to date:___________

Because of non-pregnancy related illness ☐

If yes, which?:____
Numbers of days absent to date:____________

Because of working environment ☐
If yes, why :____
Numbers of days absent to date:____________

For other reasons ☐

Multiresistant bacteria

To minimize the risk of infecting hospitalized patients or children, we kindly ask you to answer the following questions. Methicillin-resistant Staphylococcus aureus (MRSA) is a type of bacteria you can carry without being affected by them. If you have been exposed to the risk of infection, a simple examination can determine whether you are carrying these bacteria on your skin.

Have you been employed, hospitalized or had examinations/treatment in a hospital outside Denmark within the past year?: No ☐ Yes ☐ Don't know ☐

Have you ever had MRSA (Methicillin resistant Staphylococcus aureus)?
No ☐ Yes ☐ Don't know ☐

Has anyone in your nearest family had MRSA? No ☐ Yes ☐ Don't know ☐

Have any of your family ever been hospitalised in a department where there was MRSA (Methicillin resistant Staphylococcus aureus)? No ☐ Yes ☐ Don't know ☐

This pregnancy

Please describe your thoughts and wishes for this pregnancy:

____
____
____
____

Thank you for completing and returning the information sheet. This will help us to give you better advice and guidance on your pregnancy, delivery and post partum period.

Kind regards

Midwives and obstetricians at Rigshospitalet
